# No QTc prolongation with CDK 4/6 inhibitor FCN-437c: results of a concentration-QTc analysis from a dedicated study in adult healthy subjects

**DOI:** 10.3389/fphar.2024.1433663

**Published:** 2024-08-12

**Authors:** Lin Zhao, Yi Sun, Xiaoran Yang, Ling Tian, Lize Li, Fangfang Wang, Xiaoye Niu, Lei Diao, Haiyan Li

**Affiliations:** ^1^ Department of Drug Clinical Trial Center, Peking University Third Hospital, Haidian, Beijing, China; ^2^ Shanghai Fosun Pharmaceutical Development Co. Ltd., Shanghai, China; ^3^ Avanc Pharmaceutical Co. Ltd., Jinzhou, China; ^4^ Department of Cardiology and Institute of Vascular Medicine, Peking University Third Hospital, NHC Key Laboratory of Cardiovascular Molecular Biology and Regulatory Peptides, Key Laboratory of Molecular Cardiovascular Science, Ministry of Education, Beijing Key Laboratory of Cardiovascular Receptors Research, Haidian, Beijing, China

**Keywords:** FCN-437c, QT interval, concentration-QTC modeling, CDK4/6 cell cycle inhibitors, C-QT

## Abstract

Cardiotoxicity and QT interval prolongation have been a common cause of withdrawal of drugs from the market. FCN-437c is an oral, second-generation, potent, and selective CDK4/6 inhibitor for the treatment of patients with HR+/HER2- metastatic breast cancer. A single-center, double-blind, randomized, and placebo-controlled clinical study in healthy subjects was conducted to investigate the QTc prolongation potential of FCN-437c utilizing Concentration-QTc (C-QTc) modeling approach. FCN-437c was administered at doses of 300, and 400 mg with single oral administration, along with placebo, in 18 healthy subjects. Electrocardiograms (ECGs) through 24 h holter monitor and blood samples were collected. The C_max_ of 400 mg single dose in healthy subjects is similar to that from therapeutic dose 200 mg QD at steady state in patients with cancer. The 90% CI upper limit of ΔΔQTcF at the C_max_ geometric mean in both dose groups were <10 ms. It is concluded that FCN-437c has low risk of prolonging the QT interval at therapeutic dose.

**Systematic Review Registration:**

https://clinicaltrials.gov/study/NCT06290466?term=NCT06290466&rank=1, identifier [NCT06290466].

## 1 Introduction

Cyclin D-CDKs are enzyme complexes acting as critical regulators of cell cycle progression (Konecny, 2016). Cyclin-dependent kinases 4 and 6 (CDK4/6) play an essential role in cellular proliferation and are often dysregulated in breast cancer (BC), particularly in hormone-receptor (HR)-positive disease (Santamaria and Ortega, 2006; Sherr et al., 2016). The CDK4/6-cyclin D1 complex mediates cell transition into S phase via phosphorylating the retinoblastoma protein, which leads to subsequent dissociation of E2F transcription factors and induction of S-phase gene expression (Giacinti and Giordano, 2006; O’leary et al., 2016; Yu et al., 2001; Yu et al., 2006; Zhu et al., 2013). Given their essential role in cell cycle progression, CDK4/6 present promising therapeutic targets in BC with three orally-administered CDK4/6 inhibitors, i.e., palbociclib, ribociclib, and abemaciclib, approved by the FDA for the treatment of patients with HR+/HER2- metastatic BC. FCN-437c is an oral, second-generation, potent, and selective CDK4/6 inhibitor with no inhibitory activity against CDK1, CDK2, or CDK5. *In vitro* studies, FCN-437c showed inhibitory effects on cell proliferation in human BC cell lines MCF7 and MCF/ARO, which were comparable to or greater than ribociclib and palbociclib. The encouraging results of FCN-437c in HR+/HER2- advanced BC patients has been confirmed in clinical phase I (Zhang et al., 2021), phase II (Shi et al., 2023) and phase III clinical trials. FCN-437c combined with fulvestrant following disease progression on endocrine therapy in the adult patients with HR+/HER2- advanced or metastatic BC is currently under NDA review by China NMPA. And FCN-437c combined with an aromatase inhibitor as initial endocrine-based therapy was being prepared for NDA submission.

Drug induced cardiotoxicity is a major cause of market withdrawal. In the last decade of the 20th century, eight non-cardiovascular drugs were withdrawn from clinical use due to QT interval prolongation (Fermini and Fossa, 2003). Assessment of the potential of an investigational agent to cause QTc prolongation as a biomarker for ventricular tachycardia is an essential component of new drug development, as drugs that prolong the QTc interval pose an increased risk for ventricular tachycardia and sudden cardiac death. The International Council for Harmonization of Technical Requirements for Pharmaceuticals for Human Use (ICH) E14 and ICH S7B (2005) guidance recommends conducting a well-controlled, thorough QT/QTc study (TQT study) to evaluate a drug candidate’s proarrhythmic risk and its potential to delay cardiac repolarization (ICH, 2005b; ICH, 2005a), however, conducting the TQT study and achieving clinical and supratherapeutic clinical exposure can often be challenging in oncology clinical development depending upon the safety profile of the compound (Sarapa and Britto, 2008). In recent years, the ICH E14 established concentration-QTc (C-QTc) modeling as a primary analysis of QT prolongation risk (ICH, 2015). This method was outlined in further detail in the white paper described by Garnett et al. (2018).

A dedicated concentration-QTc study (NCT06290466 https://clinicaltrials.gov/study/NCT06290466?term=NCT06290466&rank=1) of FCN-437c was conducted to evaluated the QTc prolongation potential of FCN-437c. FCN-437c was administered orally at a dose of 200 mg once daily in phase III studies. PK results of the phase Ia study showed that when administered as a single dose, the exposure (C_max_ and AUC_0-∞_) of FCN-437c increased almost in proportion with doses from 50 to 450 mg. When FCN-437c was administered in multiple doses, the exposure also increased almost in proportion with doses from 50 to 200 mg. At the higher doses (200–450 mg), there appeared to be a trend of saturation. Mean accumulation ratio for AUC_0-∞_ and C_max_ ranged from 1.59 to 3.12 and from 1.24 to 1.63, respectively (Zhang et al., 2021). The geometric mean C_max_ for 450 mg single dose and C_max,ss_ for 200 mg q.d. dose were 1,528.6 ng/mL and 1,181.2 ng/mL, respectively. The geometric mean AUC_0-∞_ for 450 mg single dose and AUC_0-∞,ss_for 200 mg q.d. dose were 40,259.749 h*ng/mL and 45,360.698 h*ng/mL, respectively. Overall, the C_max_ of single dose of 400 mg could cover the C_max_ of therapeutic dose of 200 mg QD at steady state. Median time to peak FCN-437c plasma concentration (T_max_) is around 3–4 h, so the PK and ECGs sampling time points were designed to include 3 and 4 h after dosing in this C-QTc study.

## 2 Methods

### 2.1 Study design and treatment

This was a single-center, double-blind, randomized, and placebo-controlled study in healthy Chinese subjects. The primary objective was to evaluate the effects of single doses of FCN-437c on heart rate (HR)-corrected QT interval using Fridericia’s formula (QTcF) compared with placebo in healthy Chinese subjects through concentration-QTc modeling approach. The secondary objectives were to assess the effects of single doses of FCN-437c 300 and 400 mg on echocardiogram (ECG) parameters (HR, pulse rate (PR), and QRS intervals), PK, and the safety and tolerability of FCN-437c in healthy Chinese subjects.

All eligible subjects were admitted to the clinical research unit on day −1 and were domiciled in the clinical research unit until day 9. Subjects were randomized 2:1 to single oral doses of 300, or 400 mg FCN-437c or placebo following fasting for at least 10 h. Higher doses (400 mg) were administered only after completion of the lower dose (300 mg) administration, and satisfactory safety and tolerability assessments were confirmed on the fourth day post-administration.

The research was carried out at the Peking University Third Hospital. The subjects were admitted to the phase 1 ward. The study was conducted in accordance with the ethical principles communicated in the Declaration of Helsinki, ICH Good Clinical Practice guidelines, and applicable regulatory requirements and in compliance with the protocol. All subjects provided written informed consent prior to their participation in the studies.

### 2.2 Study participants

Participants were healthy Chinese males and females between the ages of 18 and 45 years, inclusive, with a body weight of ≥45 kg for females and ≥50 kg for males, body mass index of ≥19 to ≤26 kg/m^2^. Participants had to be in stable health without any evidence of cardiovascular disease, other significant medical conditions, or clinically significant laboratory test abnormalities. All subjects were required to agree to take appropriate and effective contraceptive measures from 2 weeks before screening until 6 months after administration of FCN-437c.

Key exclusion criteria included significantly abnormal findings in electrocardiogram examination (QTcF ≥ 450 ms, PR ≥ 200 ms, QRS ≥ 120 ms), or any history of organic heart disease and clinically significant electrolyte disturbances, any clinically significant physical examination, vital signs, laboratory tests and 12-ECG examination abnormalities, positivity for HBV surface antigen, HCV antibodies, *treponema pallidum* antibodies and HIV antibodies, receiving any drug or Chinese herbal medicine within 14 days before screening, or receiving any drug that affects liver metabolic enzymes or prolong the QT/QTc interval within 30 days before screening, any evidence of cardiovascular disease, other significant medical conditions, smoking within 3 months before screening, history of alcoholism (defined as > 14 units per week) within 6 months before screening or positive alcohol test during screening, history of drug abuse within 3 months before screening and pregnant or lactating women. Additional details of the complete list of inclusion and exclusion criteria can be found on clinicaltrials.gov (NCT06290466).

### 2.3 Plasma concentrations and PK analysis

Blood samples for PK analysis of FCN-437c were collected at predose and at 1, 2, 3, 4, 5, 6, 8, 12, 24, 48, 72, 120, 144, 168, and 192 h postdose. The determination of plasma concentrations of FCN-437c were performed by Fosun Eddie (Suzhou) Pharmaceutical Technology Co., Ltd., using validated LCMS methods. The lower limit of quantification was 5.00 ng/mL.

PK analysis was performed based on actual time of sample collection, using noncompartmental methods with SAS version 9.4 (SAS Institute Inc.). All plasma concentrations below the lower limit of quantification were treated as missing, except those measured rior to the T_max_, which was treated as “0”. PK parameters included C_max_, T_max_, AUC_0–t_, AUC_0–inf_, *t*_½_ and so on. AUC was estimated using the linear trapezoidal method (linear up log down). If AUC_0-t_/AUC_0-∞_< 0.8, no value of AUC_0–inf_ or *t*_½_ was reported.

### 2.4 Electrocardiogram and QTc analysis

In this study, a continuous ECG recording was performed for 25 h, starting 1 h prior to dosing. The ECG data for C-QTc analysis were collected using Mortara H12+™ Holter Monitor. During the whole study, QTc was measured blindly by two dedicated ECG technicians. And the ECG database was locked before any statistical analysis was undertaken. The 12-lead holter ECGs were extracted at the following time points: −30, −20, and −10 min prior to dosing, and 1, 2, 3, 4, 5, 6, 8, 12, and 24 h after dosing. Three electrocardiograms are extracted at each planed time point before PK sample collection. Prior to each dynamic electrocardiogram collection, the participant must maintain a supine position at least 10 min. Following the completion of the electrocardiogram sequence, a pharmacokinetics blood sample should be collected immediately.

To reduce the dependence of QT on heart rate (HR), Fridericia’s correction was used for all analyses.

QTcF=QT/RR^0.33,RR=60/HR

### 2.5 Analyses for 12-lead ECG data and C-QTc analysis

All statistical analysis of ECG data, including the C-QTc analysis, were performed using SAS version 9.4 (SAS Institute Inc.). Baseline was the average of the derived ECG intervals from the three time points prior to dosing.

The number (percentage) of participants and time points in absolute QTcF interval values ≤450 ms, >450 ms and ≤480 ms, >480 ms and ≤500 ms, and >500 ms, and changes from predose baseline of ≤30 ms, >30 and ≤60 ms, and >60 ms were summarised.

The change from baseline in QTc (∆QTcF) and the change from baseline QTc adjusted for placebo (∆∆ QTcF) were calculated using Eqs 1, 2, respectively:

$$\Delta {QTcF}_{ijk}=\bar{{QTcF}_{\text{ijk}•}}-\;{\bar{QTcF}}_{ij\left(k<0\right)•}$$ (1)

where $\bar{{QTcF}_{\text{ijk}•}}$ is measurement point k of the ith subject in group j, ${\bar{\text{QTcF}}}_{\text{ij}\left(k<0\right)•}$ is the baseline QTcF value of the ith subject, that is, the mean value of QTcF for the three times before administration.

$${\Delta \Delta \text{QTcF}}_{i,j=1,k}={\Delta \text{QTcF}}_{i,j=1,k}-{\bar{∆\text{QTcF}}}_{i,j=0,k}$$ (2)

Where i is subject, j is treatment (0 = placebo, 1 = active drug), k is measurement point. The ${\Delta \Delta \text{QTcF}}_{i,j=1,k}$ and ${\Delta \text{QTcF}}_{i,j=1,k}$ are ΔΔQTcF and ΔQTcF at measurement point k of the ith subject in FCN-437c treatment group, respectively. The ${\bar{∆\text{QTcF}}}_{i,j=0,k}$ is mean value of ΔQTcF at measurement point k of all subjects in placebo treatment group.

The C-QTc analysis was based on time-matched ∆QTcF. The analysis consisted of an exploratory step where the requisite modeling assumptions were explored followed by model development. This study used the prespecified linear mixed-effects method to establish FCN-437c drug plasma concentration and QTcF model. According to the recommendations of scientific white paper (ICH, 2015), if the 4 hypotheses (see left column of Supplementary Table S1) are met, the preset linear model can be used for analysis. The linear mixed effect model was applied using ΔQTcF as the dependent variable, which was described by the following equation:

$${\Delta \text{QTcF}}_{ijk}=\left(\theta_{0}+\eta_{0,i}\right)+\theta_{1}{TRT}_{j}+\left(\theta_{2}+\eta_{2,i}\right)C_{ijk}+\theta_{3}{TIME}_{k}+\theta_{4}\left({\text{QTcF}}_{ijk<0}-{\bar{\text{QTcF}}}_{jk<0}\right)$$ (3)

where ∆QTcF_ijk_ and C_ijk_ are the change from baseline in QTc and drug plasma concentration, respectively, at time k of the ith subject in the group j; θ_0_ is the population mean intercept in the absence of a treatment effect; θ_1_ is the fixed effect of group TRT_j_ (j = 0, placebo; j = 1, FCN-437c); θ_2_ is the population mean slope of the assumed linear association between concentration and ∆QTcF_ijk_; θ_3_ is the fixed effect of correction time factor; θ_4_ is the fixed effect of correction baseline; QTcF_ijk< 0_ is the baseline average QTcF of the ith subject in the group j; and ${\bar{\text{QTcF}}}_{jk<0}$ is the mean of QTcF at time 0 (baseline) for all subjects. The η_0,i_ and η_2,i_ are random effects between individual θ_1_ and θ_2_, respectively. It is assumed that the random effects are normally distributed with mean [0,0] and an unstructured covariance matrix G, whereas the residuals are normally distributed with mean 0 and variance R.

The optimal fitted model was used to predict population average ∆∆QTcF and its corresponding 90% two-sided confidence interval (CI) at the observed geometric mean C_max_ at each dose of FCN-437c in this study and at the clinical exposure of 200 mg in BC patients. FCN-437c would be deemed to have no clinically relevant QT effect if the upper limit of the two-sided 90% CI of the predicted effect at the observed geometric mean C_max_ was below 10 ms.

## 3 Results

### 3.1 Study population

A total of 18 participants were randomized and received FCN-437c 300 mg (n = 6), 400 mg (n = 6) or placebo (n = 6) for once. All subjects were included in the C-QTc analysis dataset. The characteristics of the subjects in the C-QTc analysis are shown in Table 1. In total this included seven female subjects and 11 male subjects. The subjects ranged in age from 20 to 43 years. Their body weights ranged from 48.3 to 79.8 kg.

**TABLE 1:** Baseline demographic characteristics.

		FCN-437c		Total
	Placebo	300 mg	400 mg	
Age (y)				
n	6	6	6	18
Mean	30.3	35.3	29.2	31.6
SD	7.92	5.16	8.08	7.29
Median	31.5	34.5	27.5	32.0
Range	20–43	28–43	21–40	20–43
Sex - n (%)				
Male	3 (50.0)	4 (66.7)	4 (66.7)	11 (61.1)
Female	3 (50.0)	2 (33.3)	2 (33.3)	7 (38.9)
Height (cm)				
n	6	6	6	18
Mean	162.75	165.42	172.00	166.72
SD	9.385	8.291	7.603	8.895
Median	162.75	165.00	175.00	165.75
Range	148.5–176.0	157.0–179.0	159.5–178.5	148.5–179.0
Body weight (kg)				
n	6	6	6	18
Mean	59.97	63.07	71.33	64.79
SD	6.444	10.896	8.207	9.556
Median	57.30	64.30	72.25	65.45
Range	53.8–69.6	48.3–77.4	60.5–79.8	48.3–79.8
BMI (kg/m^2^)				
n	6	6	6	18
Mean	22.67	22.93	24.07	23.22
SD	1.821	2.603	1.537	2.013
Median	22.65	24.35	24.90	24.30
Range	19.5–24.5	19.6–25.2	22.0–25.4	19.5–25.4

Abbreviations: BMI, body mass index.

### 3.2 Pharmacokinetics

A summary of FCN-437c PK parameters for 300mg and 400 mg is presented in Table 2, and the mean FCN-437c plasma concentration-time profiles of FCN-437c are presented in Supplementary Figure S1. After single oral doses of FCN-437c 300 and 400 mg, median T_max_ was observed at approximately 5–5.5 h postdose. There was a 1.5-fold increase in C_max_ (758.4 ng/mL for 300 mg, 1,147.4 ng/mL for 400 mg) and a 1.2-fold increase in AUC_0–∞_ (25,117.8 h*ng/mL for 300 mg, 30,653.1 h*ng/mL for 400 mg) following a 1.3-fold increase in dose from 300 mg to 400 mg. The mean t_1/2_ was similar at 300 and 400 mg, approximately 45 h.

**TABLE 2:** Summary of FCN-437c PK parameters.

Parameters (Unit)	FCN-437c 300 mg	FCN-437c 400 mg
T_max_ (h)^a^	5.0 (2.0–6.0)	5.5 (3.0–6.0)
C_max_ (ng/mL)^b^	758.4 (22.8)	1,147.4 (16.6)
AUC_0-t_ (h*ng/mL)^b^	24,154.8 (14.1)	29,839.8 (9.7)
AUC_0-∞_ (h*ng/mL)^b^	25,117.8 (14.5)	30,653.1 (9.8)
t_1/2_ (h)^b^	45.021 (22.5)	44.877 (10.2)
CL/F (L/h)^b^	11.944 (14.5)	13.049 (9.8)
Vz/F (L)^b^	775.763 (21.2)	844.857 (13.7)

Abbreviations: AUC_0-t_, area under the curve from 0 h to the time at which the lowest plasma concentration can be detected; AUC_0-∞_, area under the curve extrapolated from 0 h to infinity; C_max_, peak plasma concentration; CL/F, apparent clearance; Vz/F, apparent distribution volume; T_max_, peak time; t_1/2_, terminal elimination half life.

^a^ Median (min-max).

^b^ Geometric mean (geometric CV%).

### 3.3 ECG results

A categorized summary of QTcF and ΔQTcF was performed based on dose groups and sampling points. As shown in Supplementary Table S2, no outliers were detected in QTcF (i.e., >450 and ≤480 ms, >480 and ≤500 ms, or> 500 ms when not present at baseline) in any treatment group. Similarly, no outliers were found in ΔQTcF (i.e., >30 and ≤60 ms or >60 ms) in either the FCN-437c or placebo groups.

### 3.4 Safety

A total of 25 treatment emergent adverse events (TEAEs) were reported by 14 (77.8%) of subjects. The incidence of TEAEs was 100.0% (6/6), 50.0% (3/6) and 83.3% (5/6) for 300 mg, 400 mg and placebo, respectively (Supplementary Table S3). The 300 mg group study was conducted during the COVID-19 pandemic, caused seven subjects reporting AEs due to COVID-19 infections, two of them from the placebo group and five from the FCN-437c 300 mg group.

Most TEAEs related to the study drug mainly include decreases in hemoglobin and abnormal liver function, which were similar to the common adverse reactions reported in previous studies of CDK4/6 inhibitors, and the severity were grade 1, with incidence rates of 50.0% (3/6), 66.7% (4/6), and 33.3% (2/6) for placebo, 300 mg, and 400 mg, respectively. There was no relationship between incidence of TEAEs and doses of FCN-437c. Importantly, there were no other suspicious cardiovascular adverse events, no serious or severe AEs and no withdrawals due to AEs in this study.

### 3.5 Concentration-QTc modeling

**FIGURE 1:**
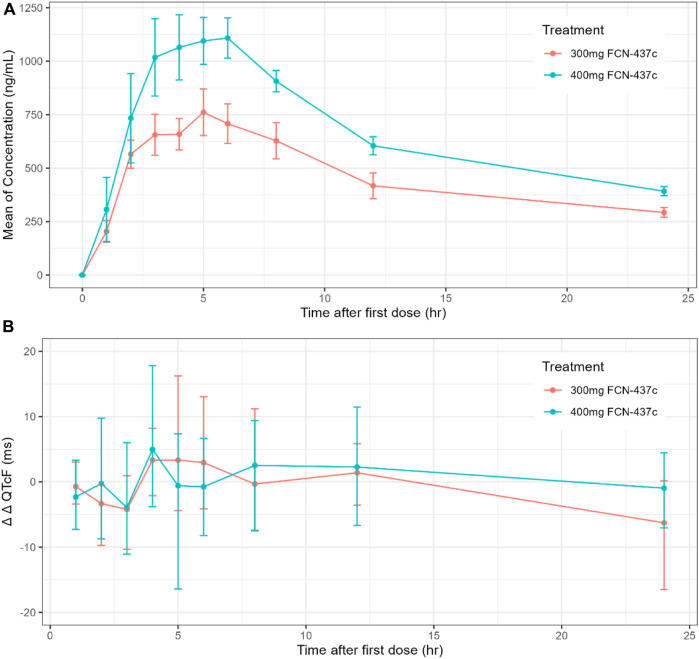
**(A)** Time course of mean and 90% CI drug concentration; **(B)** Time course of mean and 90% CI ΔΔQTcF.

**FIGURE 2:**
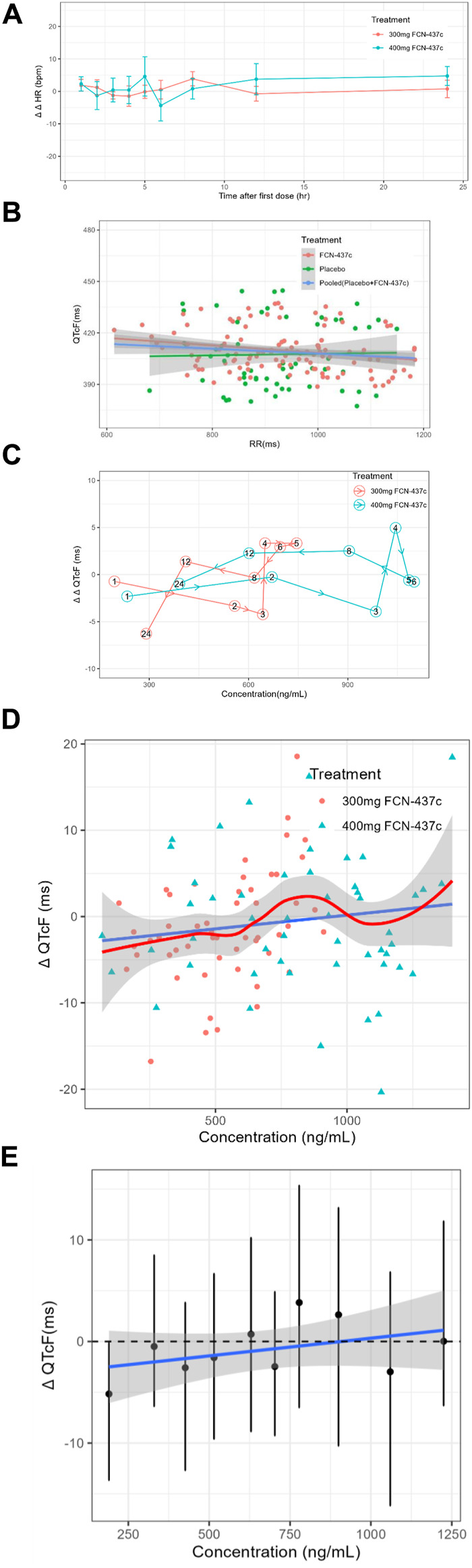
**(A)** The ΔΔHR-time curves of all cohorts (Bars show 90% confidence interval). **(B)** Scatterplot of QTcF and RR intervals by treatment. **(C)** Mean ΔΔQTcF and concentration connected in temporal order by dose. **(D)** Scatter plot of paired ΔQTc and concentration data with loess smooth line and 95% confidence intervals (shading) and linear regression line (solid line). **(E)** Quantile plot of paired ΔQTcF and concentration data. The black dots with vertical bars denote the observed mean (90% CI) ΔQTcF at the median FCN-437c plasma concentration within each decile. The solid blue line within the gray shaded area denotes the model-predicted mean (90% CI) ΔQTcF.

The mean concentration and QTc profiles by dose level and time are shown in Figure 1. The plasma concentration data and QTc data of 18 subjects were combined for model development. A C-QTc model was established with the plasma concentration as the independent variable and ΔQTcF as the dependent variable. When a research hypothesis is established, the preset linear model was used to describe the relationship between plasma concentration and QTcF (Supplementary Table S1):

• Hypothesis 1, because the HR changes on 300 and 400 mg FCN-437c closely followed the diurnal pattern seen with placebo [with very minimal differences in the mean change from baseline (ΔHR)], and mean placebo-corrected change from baseline (ΔΔHR) for FCN-437c was less than ± 5 bpm at all postdose time points (ΔΔHR; Figure 2A), it can be determined that FCN-437c has no effect on the HR of subjects;
• Hypothesis 2, there was no significant correlation between QTcF and RR, as shown in Figure 2B;
• Hypothesis 3, the T_max_ of ΔΔQTcF in both the 300 and 400 mg FCN-437c groups was 4 h, and the T_max_ of plasma concentration of the 300 and 400 mg FCN-437c groups was about 5–6 h. According to the result of correlation analysis between the mean ΔΔQTcF and concentration connected in temporal order (Figure 2C), there is no delay in judging the change in plasma concentration and QTcF;
• Hypothesis 4, there is a linear relationship between plasma concentration and QTcF, as shown in Figures 2D, E.

Based on a review of these data, it was concluded that this study meets the 4 research hypotheses in Supplementary Table S1 and can be analyzed using a preset linear model (Formula 3). Standard goodness of fit (GOF) plots did not show any significant signs of model misspecification (Supplementary Figure S2). The final model estimates are presented in Table 3. The estimated population slope of the C-QTc relationship was 0.003 ms (90% CI, −0.002–0.008 ms) per ng/mL, with an intercept of 1.072 ms (90% CI, −2.021–4.165 ms). In addition, since the value of zero was contained within the upper and lower limits of the 90% CI of the slope, the slope was not statistically significant from 0.

**TABLE 3:** Final model parameters and validation of subjects.

Parameters	Estimate	90% CI
Intercept ( $\theta_{0}$ ), ms	1.072	[−2.021; 4.165]
Treatment ( $\theta_{1}$ ), ms	−2.268	[−6.401; 1.866]
Time ( $\theta_{3}$ ), ms		
Time effect (1 h), ms	−3.268	[−6.008; −0.527]
Time effect (2 h), ms	−3.369	[−6.226; −0.511]
Time effect (3 h), ms	0.785	[−2.276; 3.845]
Time effect (4 h), ms	1.525	[−1.564; 4.615]
Time effect (5 h), ms	−0.437	[−3.638; 2.764]
Time effect (6 h), ms	−3.010	[−6.174; 0.153]
Time effect (8 h), ms	−5.724	[−8.702; −2.746]
Time effect (12 h), ms	−2.153	[−4.946; 0.641]
Time effect (24 h), ms	0.000	
Slope (θ_2_), ms per concentration unit	0.003	[−0.002; 0.008]
Baseline covariate (θ_4_), ms	0.043	[−0.067; 0.152]
Variance components	Estimate	
Intercept ( $\eta_{0,i}$ ), ms^2^	8.17801	
Concentration ( $\eta_{2,i}$ ), unit^2^	0.00002	
Residual variance, ms^2^	23.42036	

**FIGURE 3:**
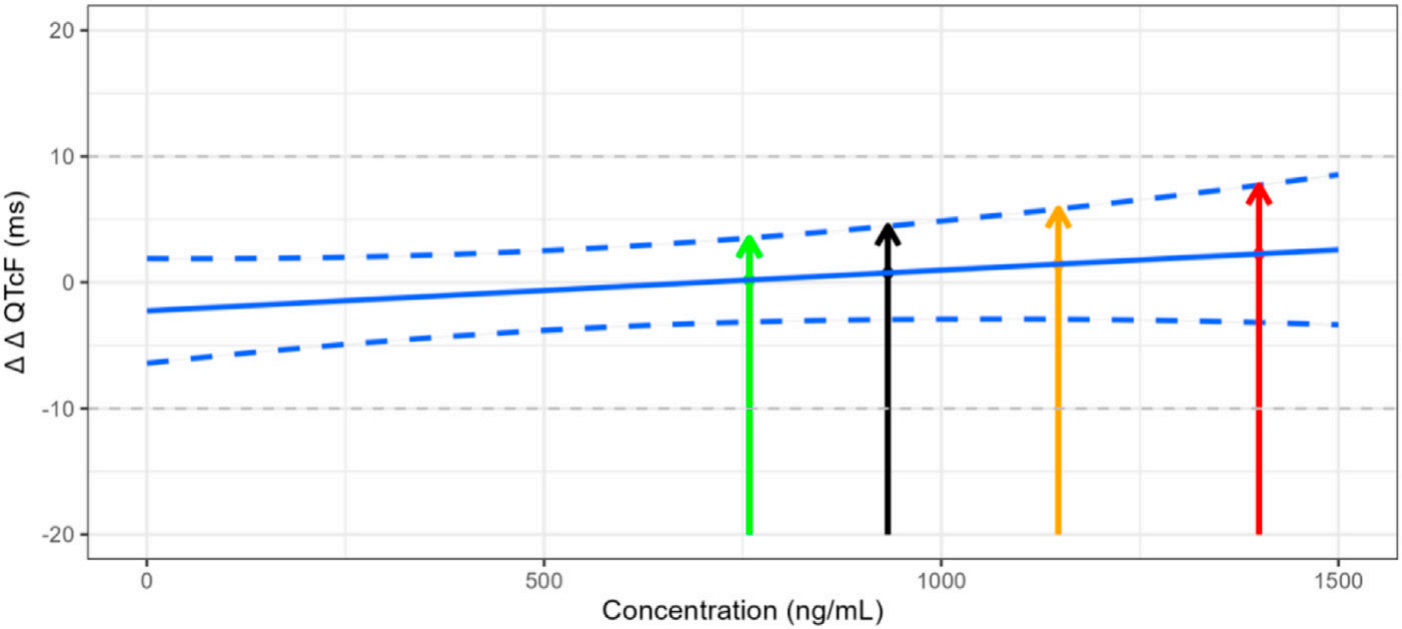
Trend of plasma concentration versus ΔΔQTcF. Note: The green line represents the ΔΔQTcF of 0.183 ms for the geometric mean C_max_ of 758.352 ng/mL in the 300 mg FCN-437c group, with the upper limit 90% confidence interval of 3.508 ms. The black line represents the ΔΔQTcF of 0.747 ms for the geometric mean C_max_ of 932.816 ng/mL in all subject received FCN-437c in this study, with the upper limit 90% confidence interval of 4.465 ms. The orange line represents the ΔΔQTcF of 1.440 ms for the geometric mean C_max_ of 1,147.417 ng/mL in the 400 mg FCN-437c group, with an upper limit 90% confidence interval of 5.867 ms. The red line represents the ΔΔQTcF of 2.257 ms for the observed maximum C_max_ of 1,400 ng/mL in this study, with the upper limit 90% confidence interval of 7.705 ms.

The QTcF at the geometric mean C_max_ of each dose group and its 2-sided 90% CI were calculated using the model formula. The results are shown in Table 4 and Figure 3. The upper limit of the 90% CI for ΔΔQTcF at the geometric mean C_max_ in each dose group was less than 10 ms. Even when the C_max_ reached 1,400 ng/mL, the corresponding ΔΔQTcF (90% CI upper limit) was 2.257 (7.705) ms, indicating that FCN-437c did not cause QTc prolongation in this study.

**TABLE 4:** Predicted ΔΔQTcF interval at geometric mean peak FCN-437c concentration.

Treatment	Geometric mean C_max_, ng/mL	Predicted (upper 90% CI) ΔΔQTcF, ms
300 mg and 400 mg (Pooled)	932.8	0.747(4.465)
300 mg	758.4	0.183(3.508)
400 mg	1,147.4	1.440(5.867)
300 mg and 400 mg (Pooled)	1,400^a^	2.257(7.705)

^a^ The observed maximum C_max_ in this study.

## 4 Discussion

This is the first published study focusing on the QTc prolongation risk of CDK4/6 inhibitor FCN-437c. Based on the 2022 revision of the ICH E14/S7B Q&A 6.1, the risk of investigational drugs causing QT interval prolongation and potential arrhythmia could be evaluated by combining the results of preclinical *in vivo* and *in vitro* research at the highest clinical exposure, along with alternative clinical QT studies, if a thorough QT study is not feasible. Considering the preclinical results and oncologic indication, this alternative concentration-QTc study with two dose levels and placebo control was designed and conducted in healthy subjects to evaluate the QTc prolongation potential of FCN-437c at therapeutic exposure.

C-QTc modeling approach has gradually become common primary analysis during small molecule oncology development since the publication of ICH E14 and the Q&A documents, and the scientific white paper (Cohen-Rabbie et al., 2021). To enhance the implementation of C-QTc in clinical drug development, the white paper provided recommendations around the design of studies and data collection for C-QTc analysis, as well as a standardized, pre-specified, statistical C-QTc model. However, the unique and complex nature of the clinical development of anticancer drugs, including the quick pace of development, differences in trial design, co-medications, and risk–benefit profile in the face of life-threatening disease, may make it impossible to meet standards for C-QTc in oncology. These uncertain elements of C-QTc evaluation may lead to inadequate implementation of C-QTc in oncology programmes, thereby potentially compromising early identification of clinical QT safety risks and compliance with regulatory requirements. Moreover, it may compromise cost-efficiency and patient burden as additional studies (e.g., dedicated QT study) and/or intensive ECG collection (e.g., in phase 2 or 3) may be required. Among the small-molecule new drug applications (NDAs) approved by FDA in oncology between 2011 and 2019, the concentration-QTc modeling approach (studies in which QT was not the primary objective) was the most common approach (59%), followed by the TQT and the dedicated QT studies (20% and 21%, respectively) (Cohen-Rabbie et al., 2021).

Although QTc assessment has significant challenges in the development of anticancer drugs, this study was deliberately designed to provide more accurate QTc prolongation assessment for FCN-437c. Considering that between study differences and potential bias when pooling two or more studies, a dedicated QT study was designed to reduce the bias caused by differences in health status and concomitant medications, as well as in study conduct or ECG acquisition or analysis (Ferber et al., 2017; Garnett et al., 2018). The supratherapeutic exposure requirement for QT assessment in the absence of positive control (at least 2-fold the highest clinically relevant drug exposure) is often loosened in oncology because the therapeutic exposure is often close to the maximum tolerated dose (Garnett et al., 2018). This study was advantageous in terms of the placebo-controlled design. The lack of placebo subjects in oncology trials typically requires that inferences from a C-QTc analysis are drawn based on baseline-corrected QTc (i.e., ΔQTc), rather than baseline-corrected, placebo-corrected QTc (ΔΔQTc). In addition, the lack of placebo data introduces diurnal fluctuation in QTc as a potential confounding factor for drug effect on QTc intervals. Although the collection of time-matched baseline (i.e., at the same time points to be collected post-dose) in order to account for diurnal fluctuation has been implemented for C-QT analysis of single-arm trials, there are still several limitations such as mean QTc interval between pre-dose and post-dose of 24 h is the same may not hold true in the clinical setting (Orihashi et al., 2021). With 200 mg having been the maximum FCN-437c dose tested in healthy volunteers prior to this study, an assessment of 300 mg was performed first in healthy volunteers. Subsequently, if the 300 mg dosage was well-tolerated, the study would then proceed with the 400 mg dosage.

Eighteen healthy subjects were enrolled in the study. Simulations demonstrated that a sample of 18 participants (12 on active, 6 on placebo) would have enough study power to exclude a small QTc effect (10 msec) by using C-QTc analysis (Ferber et al., 2015). To ensure the high-quality Holter data, ECG data were obtained/extracted from standardized 12-lead 24-h Holter ECG recordings after at least 10 min rest following a predefined algorithm defined for the manual adjudication of the ECG intervals. As such, triplicates of good quality (each recording interval is approximately 1 min) nonoverlapping ECGs were extracted from Holter within a 5-min time window before the PK sampling was performed. The same cardiologist read all the ECGs from a given participant and was blinded to ECG recording time, treatment, and participant identification. Thus, the quality of ECG records was qualified to support the C-QTc analysis.

Following multiple-dose administrations for 21 consecutives days of FCN-437c 200 mg once daily in female patients with ER+ and HER2- advanced breast cancer, the geometric mean C_max,ss_ was 1,181 ng/mL. The geometric mean C_max_ of healthy subjects received single dose of FCN-437c 300mg and 400 mg in this study was 758.4 ng/mL and 1,147.4 ng/mL, respectively. The maximum C_max_ in this study reached 1,400 ng/mL, illustrating that the exposure of FCN-437c 400 mg in healthy subjects could cover the steady state exposure in breast cancer patients who received multiple 200 mg therapeutic doses.

Currently, the food effect study is being conducted to evaluate the impact of a high-fat, high-calorie meal on the pharmacokinetic and safety profiles of single-dose FCN-437c in healthy adult subjects. FCN-437c is a biopharmaceutics classification system (BCS) class I drug with high solubility and high gastrointestinal permeability which is less likely to be affected by the physiological environment in the gastrointestinal tract. Therefore, it is hypothesized that FCN-437c’s absorption is less likely to be affected by food ingestion (no food effects) (Benet, 2013). Due to the absence of clinical studies in special populations (hepatic or renal impairment), the population pharmacokinetics analysis was conducted to assess the influence of hepatic impairment and renal impairment, the results indicated that mild hepatic or renal impairments do not significantly affect the exposure of FCN-437c. FCN-437c is a sensitive CYP3A substrate, it may lead to potential drug–drug interactions when FCN-437c is used in combination with inhibitors or inducers of CYP3A. Considering the hematological toxicity associated with FCN-437c, the dose of FCN-437c would be reduced to achieve the exposure of therapeutic dose when combined with CYP3A4 inhibitors once this speculation is confirmed by the results of the ongoing DDI study. Hence, the highe plasma concentrations resulting from a drug-drug interaction is not likely significant. As discussed in the ICH E14/S7B Q&A 5.1 (ICH, 2022), if the high clinical exposure has been achieved in the clinical ECG assessment, but a sufficient multiple has not been obtained (e.g., for reasons of safety or tolerability, saturating absorption, etc.), then a nonclinical integrated risk assessment can be used as supplementary evidence.

A wide range of exposure up to ∼ 1,400 ng/mL was observed in this study. Based on the C-QTc analysis for FCN-437c, an effect on the QTc interval exceeding 10 msec can be excluded within this observed plasma concentration range. Even when the C_max_ reached 1,400 ng/mL, the corresponding ΔΔQTcF (90% CI upper limit) was only 2.257 (7.705) ms. In addition, given that the slope of C-QTc relationship is not significant from 0 (the estimated mean slope was 0.003 ms (90% CI, −0.002–0.008 ms) per ng/mL), it seems very unlikely that FCN-437c will cause concerning QTc prolongation at clinically relevant plasma concentrations. Finally, the ΔΔQTcF corresponding to the geometric mean steady-state C_max_ of 1,181 ng/mL at the maximum therapeutic dose of 200 mg administered continuously in the target indicated patients was predicted, the corresponding ΔΔQTcF was 1.275 ms, and according to Figure 3, we can find that the upper limit of the 90% confidence interval was less than 10 ms.

The cardiovascular safety profile for FCN-437c has been assessed in both *in vitro* and *in vivo* preclinical studies. In the *in vitro* human ether-à-go-go related gene (hERG) ion channel assay, an inhibitory effect on hERG channels was noted with half-maximal inhibitory concentration of 38.9 μM. This represents a significant safety margin of approximately 617-fold, based on the clinical steady-state unbound C_max_ of 59.05 ng/mL (0.063 μM) at the recommended phase III dose of 200 mg once daily. Additionally, no abnormal electrocardiographic findings were attributable to the administration of FCN-437c in the standard cardiovascular telemetry study in beagle dogs (data on file). As the result of these nonclinical studies, the potential risk of QT interval prolongation in humans due to FCN-437c is considered to be low.

This study has several limitations. First, a sufficient multiple of the “high clinical scenario” exposure was not achieved (usually 2-fold) because the tolerability concerns of FCN-437c, such as exposure-dependent hematological toxicity, limited the supra-exposure in healthy volunteers. Second, the plasma concentrations observed in this C-QTc study may not substantially exceed drug concentrations in patients potentially increased by intrinsic or extrinsic factors, such as CYP3A4 inhibitors, so adjust the dosage of FCN-437c may be necessary to avoid increased safety risks due to the comedication. Last, considering multiple factors that can induce QT prolongation among cancer patients, such as the presence of multiple coexisting risk factors (hypothyroidism congenital long QT syndrome, left ventricular dysfunction, myocardial ischemia), the concomitant treatments, various side effects, and finally kidney failure, liver dysfunction, and poorly controlled diabetes (Teomete et al., 2024), there are certain limitations in the cardiotoxicity assessment of cancer patients using the C-QTc study results of healthy volunteers. Therefore, we can only conclude that the therapeutic exposure of FCN-437c has no effect on the cardiac QT interval of healthy adults at therapeutic exposure, and its cardiotoxicity in patients with comedication and severe conditions needs to be further explored.

In conclusion, single doses of FCN-437c 300 and 400 mg did not cause clinically significant prolongation of the QTc interval in healthy volunteers with predicted mean ΔΔQTcF <10 msec by concentration-QTc modeling. It can be concluded that FCN-437c, at the therapeutic dose of 200 mg once daily, is unlikely to cause QTc prolongation.

## Data Availability

The raw data supporting the conclusions of this article will be made available by the authors, without undue reservation.
