# Supplementary material for: No QTc prolongation with CDK 4/6 inhibitor FCN-437c: results of a concentration-QTc analysis from a dedicated study in adult healthy subjects
Source: Front Pharmacol. 2024 Aug 12;15:1433663. doi: 10.3389/fphar.2024.1433663 (PMC11346245; doi:10.3389/fphar.2024.1433663)
Supplement: Supplementary file 1 [file DataSheet1.docx]

**Table S1.** Factors to be considered in evaluating the cardiac safety of drugs with the exemption of TQT from the C-QTc model

| **Hypothesis** | **Validation result** |
| --- | --- |
| Hypothesis 1: The drug does not affect subjects’ heart rate. | Placebo-corrected change-from-baseline heart rate (ΔΔHR) of all subjects were between ±10 ms shown in Figure 2a. The unit is bpm. |
| Hypothesis 2: QTcF is not related to HR. | There was no significant correlation between QTcF and RR shown in Figure 2b. |
| Hypothesis 3: There is no hysteresis between concentration and ΔQTcF. | The difference value of T_max_ of plasma concentration and ΔQTcF in group 300 mg, 400 mg were within 1 hour. A time course of ΔΔQTcF showing concordance with PK shown in Figure 2c. |
| Hypothesis 4: Plasma concentration is linear with ΔQTcF. | The shape of concentration VS ΔQTcF did not show nonlinearity shown in Figure 2d and Figure 2e. |

**Table S2.** Summary of QTcF and ΔQTcF

|  | | | n (%) | | | |
| --- | --- | --- | --- | --- | --- | --- |
|  | | | | FCN-437c | |  |
| Timepoint | Variable | Category | Placebo | 300mg | 400mg | Pooled |
| 1h | QTcF | ≤450 ms | 6 (100.0) | 6 (100.0) | 6 (100.0) | 18 (100.0) |
|  |  | >450 ms &≤480 ms | 0 ( 0.0) | 0 ( 0.0) | 0 ( 0.0) | 0 ( 0.0) |
|  |  | >480 ms &≤500 ms | 0 ( 0.0) | 0 ( 0.0) | 0 ( 0.0) | 0 ( 0.0) |
|  |  | >500 ms | 0 ( 0.0) | 0 ( 0.0) | 0 ( 0.0) | 0 ( 0.0) |
|  | | | | | | |
|  | ΔQTcF | ≤30 ms | 6 (100.0) | 6 (100.0) | 6 (100.0) | 18 (100.0) |
|  |  | >30ms &≤60 ms | 0 ( 0.0) | 0 ( 0.0) | 0 ( 0.0) | 0 ( 0.0) |
|  |  | >60 ms | 0 ( 0.0) | 0 ( 0.0) | 0 ( 0.0) | 0 ( 0.0) |
|  | | | | | | |
| 2h | QTcF | ≤450 ms | 6 (100.0) | 6 (100.0) | 6 (100.0) | 18 (100.0) |
|  |  | >450 ms &≤480 ms | 0 ( 0.0) | 0 ( 0.0) | 0 ( 0.0) | 0 ( 0.0) |
|  |  | >480 ms &≤500 ms | 0 ( 0.0) | 0 ( 0.0) | 0 ( 0.0) | 0 ( 0.0) |
|  |  | >500 ms | 0 ( 0.0) | 0 ( 0.0) | 0 ( 0.0) | 0 ( 0.0) |
|  | | | | | | |
|  | ΔQTcF | ≤30 ms | 6 (100.0) | 6 (100.0) | 6 (100.0) | 18 (100.0) |
|  |  | >30ms &≤60 ms | 0 ( 0.0) | 0 ( 0.0) | 0 ( 0.0) | 0 ( 0.0) |
|  |  | >60 ms | 0 ( 0.0) | 0 ( 0.0) | 0 ( 0.0) | 0 ( 0.0) |
|  | | | | | | |
| 3h | QTcF | ≤450 ms | 6 (100.0) | 6 (100.0) | 6 (100.0) | 18 (100.0) |
|  |  | >450 ms &≤480 ms | 0 ( 0.0) | 0 ( 0.0) | 0 ( 0.0) | 0 ( 0.0) |
|  |  | >480 ms &≤500 ms | 0 ( 0.0) | 0 ( 0.0) | 0 ( 0.0) | 0 ( 0.0) |
|  |  | >500 ms | 0 ( 0.0) | 0 ( 0.0) | 0 ( 0.0) | 0 ( 0.0) |
|  | | | | | | |
|  | ΔQTcF | ≤30 ms | 6 (100.0) | 6 (100.0) | 6 (100.0) | 18 (100.0) |
|  |  | >30ms &≤60 ms | 0 ( 0.0) | 0 ( 0.0) | 0 ( 0.0) | 0 ( 0.0) |
|  |  | >60 ms | 0 ( 0.0) | 0 ( 0.0) | 0 ( 0.0) | 0 ( 0.0) |
|  | | | | | | |
| 4h | QTcF | ≤450 ms | 6 (100.0) | 6 (100.0) | 6 (100.0) | 18 (100.0) |
|  |  | >450 ms &≤480 ms | 0 ( 0.0) | 0 ( 0.0) | 0 ( 0.0) | 0 ( 0.0) |
|  |  | >480 ms &≤500 ms | 0 ( 0.0) | 0 ( 0.0) | 0 ( 0.0) | 0 ( 0.0) |
|  |  | >500 ms | 0 ( 0.0) | 0 ( 0.0) | 0 ( 0.0) | 0 ( 0.0) |
|  | | | | | | |
|  | ΔQTcF | ≤30 ms | 6 (100.0) | 6 (100.0) | 6 (100.0) | 18 (100.0) |
|  |  | >30ms &≤60 ms | 0 ( 0.0) | 0 ( 0.0) | 0 ( 0.0) | 0 ( 0.0) |
|  |  | >60 ms | 0 ( 0.0) | 0 ( 0.0) | 0 ( 0.0) | 0 ( 0.0) |
|  | | | | | | |
| 5h | QTcF | ≤450 ms | 6 (100.0) | 6 (100.0) | 6 (100.0) | 18 (100.0) |
|  |  | >450 ms &≤480 ms | 0 ( 0.0) | 0 ( 0.0) | 0 ( 0.0) | 0 ( 0.0) |
|  |  | >480 ms &≤500 ms | 0 ( 0.0) | 0 ( 0.0) | 0 ( 0.0) | 0 ( 0.0) |
|  |  | >500 ms | 0 ( 0.0) | 0 ( 0.0) | 0 ( 0.0) | 0 ( 0.0) |
|  | | | | | | |
|  | ΔQTcF | ≤30 ms | 6 (100.0) | 6 (100.0) | 6 (100.0) | 18 (100.0) |
|  |  | >30ms &≤60 ms | 0 ( 0.0) | 0 ( 0.0) | 0 ( 0.0) | 0 ( 0.0) |
|  |  | >60 ms | 0 ( 0.0) | 0 ( 0.0) | 0 ( 0.0) | 0 ( 0.0) |
|  |  |  |  |  |  |  |
| 6h | QTcF | ≤450 ms | 6 (100.0) | 6 (100.0) | 6 (100.0) | 18 (100.0) |
|  |  | >450 ms &≤480 ms | 0 ( 0.0) | 0 ( 0.0) | 0 ( 0.0) | 0 ( 0.0) |
|  |  | >480 ms &≤500 ms | 0 ( 0.0) | 0 ( 0.0) | 0 ( 0.0) | 0 ( 0.0) |
|  |  | >500 ms | 0 ( 0.0) | 0 ( 0.0) | 0 ( 0.0) | 0 ( 0.0) |
|  |  |  |  |  |  |  |
|  | ΔQTcF | ≤30 ms | 6 (100.0) | 6 (100.0) | 6 (100.0) | 18 (100.0) |
|  |  | >30ms &≤60 ms | 0 ( 0.0) | 0 ( 0.0) | 0 ( 0.0) | 0 ( 0.0) |
|  |  | >60 ms | 0 ( 0.0) | 0 ( 0.0) | 0 ( 0.0) | 0 ( 0.0) |
|  | | | | | | |
| 8h | QTcF | ≤450 ms | 6 (100.0) | 6 (100.0) | 6 (100.0) | 18 (100.0) |
|  |  | >450 ms &≤480 ms | 0 ( 0.0) | 0 ( 0.0) | 0 ( 0.0) | 0 ( 0.0) |
|  |  | >480 ms &≤500 ms | 0 ( 0.0) | 0 ( 0.0) | 0 ( 0.0) | 0 ( 0.0) |
|  |  | >500 ms | 0 ( 0.0) | 0 ( 0.0) | 0 ( 0.0) | 0 ( 0.0) |
|  | | | | | | |
|  | ΔQTcF | ≤30 ms | 6 (100.0) | 6 (100.0) | 6 (100.0) | 18 (100.0) |
|  |  | >30ms &≤60 ms | 0 ( 0.0) | 0 ( 0.0) | 0 ( 0.0) | 0 ( 0.0) |
|  |  | >60 ms | 0 ( 0.0) | 0 ( 0.0) | 0 ( 0.0) | 0 ( 0.0) |
|  | | | | | | |
| 12h | QTcF | ≤450 ms | 6 (100.0) | 6 (100.0) | 5 ( 83.3) | 17 ( 94.4) |
|  |  | >450 ms &≤480 ms | 0 ( 0.0) | 0 ( 0.0) | 0 ( 0.0) | 0 ( 0.0) |
|  |  | >480 ms &≤500 ms | 0 ( 0.0) | 0 ( 0.0) | 0 ( 0.0) | 0 ( 0.0) |
|  |  | >500 ms | 0 ( 0.0) | 0 ( 0.0) | 0 ( 0.0) | 0 ( 0.0) |
|  |  | Missing | 0 ( 0.0) | 0 ( 0.0) | 1 ( 16.7) | 1 ( 5.6) |
|  |  |  |  |  |  |  |
|  | ΔQTcF | ≤30 ms | 6 (100.0) | 6 (100.0) | 5 ( 83.3) | 17 ( 94.4) |
|  |  | >30ms &≤60 ms | 0 ( 0.0) | 0 ( 0.0) | 0 ( 0.0) | 0 ( 0.0) |
|  |  | >60 ms | 0 ( 0.0) | 0 ( 0.0) | 0 ( 0.0) | 0 ( 0.0) |
|  |  | Missing | 0 ( 0.0) | 0 ( 0.0) | 1 ( 16.7) | 1 ( 5.6) |
|  | | | | | | |
| 24h | QTcF | ≤450 ms | 6 (100.0) | 6 (100.0) | 5 ( 83.3) | 17 ( 94.4) |
|  |  | >450 ms &≤480 ms | 0 ( 0.0) | 0 ( 0.0) | 0 ( 0.0) | 0 ( 0.0) |
|  |  | >480 ms &≤500 ms | 0 ( 0.0) | 0 ( 0.0) | 0 ( 0.0) | 0 ( 0.0) |
|  |  | >500 ms | 0 ( 0.0) | 0 ( 0.0) | 0 ( 0.0) | 0 ( 0.0) |
|  |  | Missing | 0 ( 0.0) | 0 ( 0.0) | 1 ( 16.7) | 1 ( 5.6) |
|  |  |  |  |  |  |  |
|  | ΔQTcF | ≤30 ms | 6 (100.0) | 6 (100.0) | 5 ( 83.3) | 17 ( 94.4) |
|  |  | >30ms &≤60 ms | 0 ( 0.0) | 0 ( 0.0) | 0 ( 0.0) | 0 ( 0.0) |
|  |  | >60 ms | 0 ( 0.0) | 0 ( 0.0) | 0 ( 0.0) | 0 ( 0.0) |
|  |  | Missing | 0 ( 0.0) | 0 ( 0.0) | 1 ( 16.7) | 1 ( 5.6) |
|  | | | | | | |
|  | | | | | | |

**Table S3.** Number (%) of healthy volunteers with treatment-emergent adverse events

|  | Placebo  (N=6) | 300mg FCN-437c  (N=6) | 400mg FCN-437c  (N=6) | Total  (N=18) |
| --- | --- | --- | --- | --- |
|  | n (%) | n (%) | n (%) | n (%) |
| Any adverse event | 5 (83.3) | 6 (100.0) | 3 (50.0) | 14 (77.8) |
| TEAE | 5 (83.3) | 6 (100.0) | 3 (50.0) | 14 (77.8) |
| FCN-437c related TEAE | 3 (50.0) | 2 (33.3) | 2 (33.3) | 7 (38.9) |
| Grade 3/4 TEAE | 0.0 (0.0) | 0.0 (0.0) | 0.0 (0.0) | 0.0 (0.0) |
| Grade 3/4 FCN-437c related TEAE | 0.0 (0.0) | 0.0 (0.0) | 0.0 (0.0) | 0.0 (0.0) |
| SAE | 0.0 (0.0) | 0.0 (0.0) | 0.0 (0.0) | 0.0 (0.0) |
| FCN-437c related SAE | 0.0 (0.0) | 0.0 (0.0) | 0.0 (0.0) | 0.0 (0.0) |
| TEAE leading to withdraw | 0.0 (0.0) | 0.0 (0.0) | 0.0 (0.0) | 0.0 (0.0) |
| TEAE leading to death | 0.0 (0.0) | 0.0 (0.0) | 0.0 (0.0) | 0.0 (0.0) |

**Figure S1** Mean + SD FCN-437c plasma concentration by time and dose. Red line, FCN-437c 300 mg (n = 6); blue line, FCN-437c 400 mg (n = 6).

.


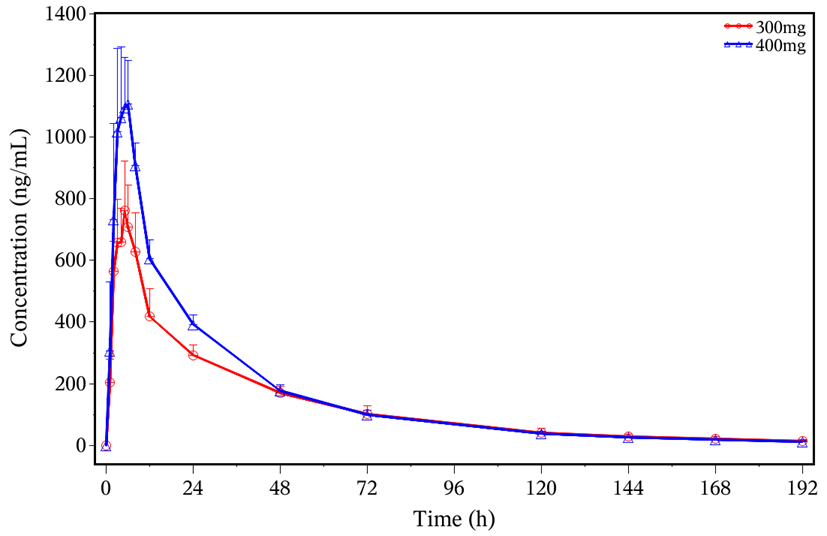


**Figure S2** Goodness of fit plots for the final C-QTc model

| **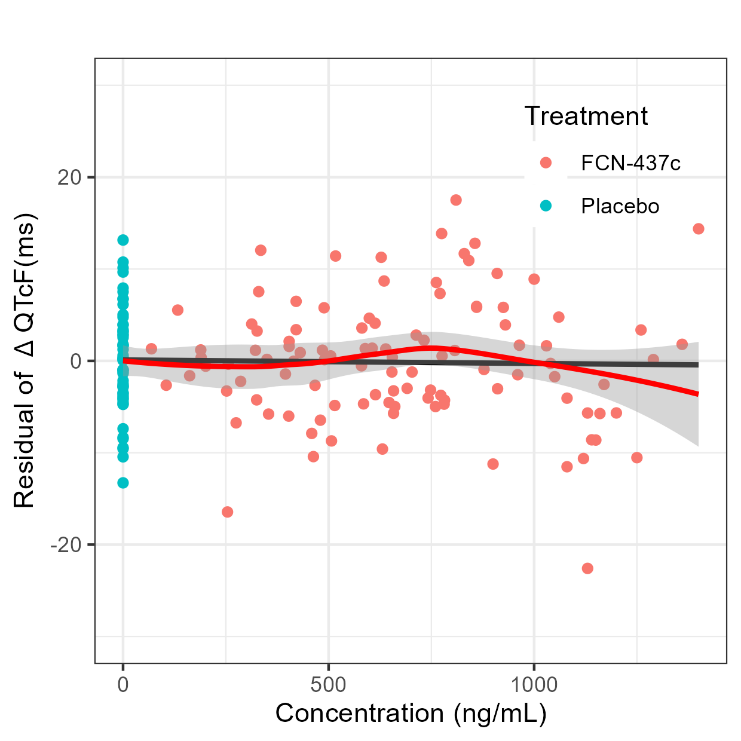** | **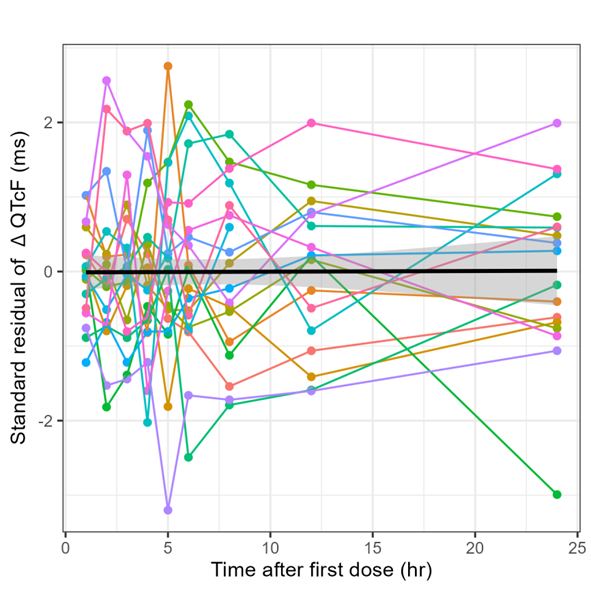** |
| --- | --- |
| 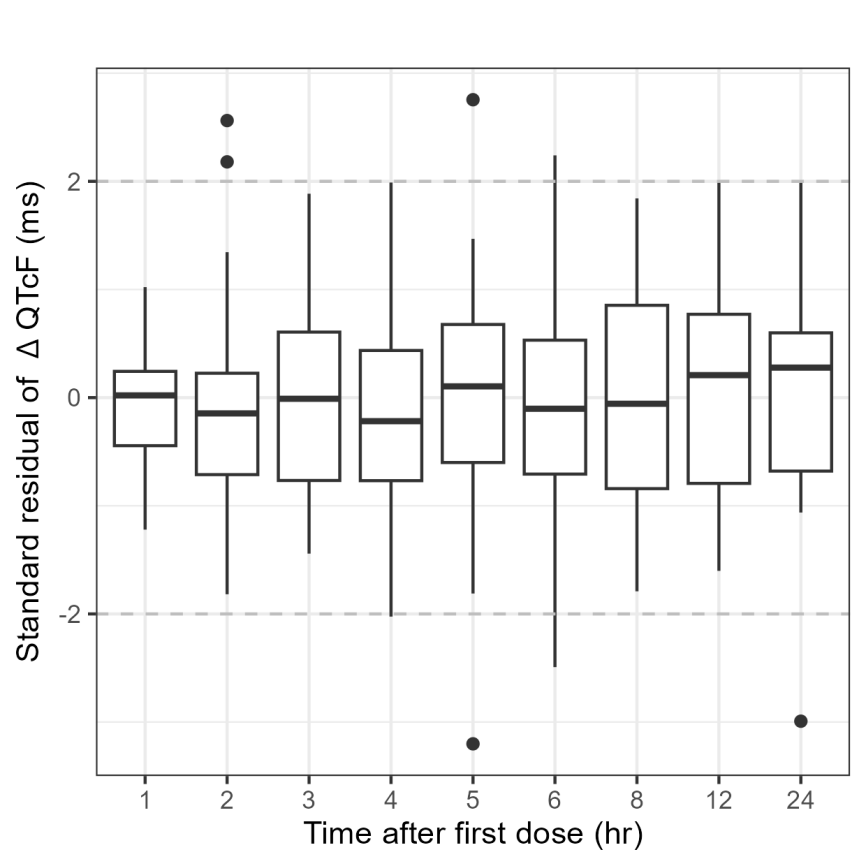 | **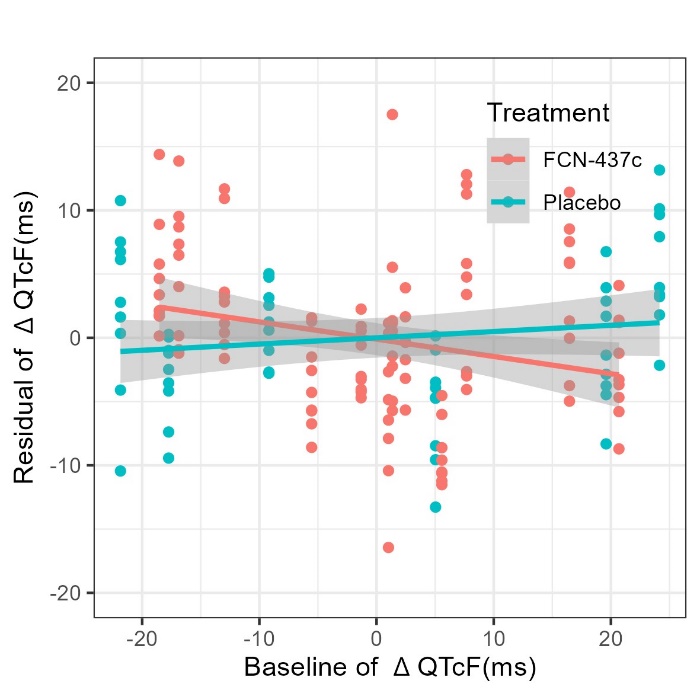** |
| 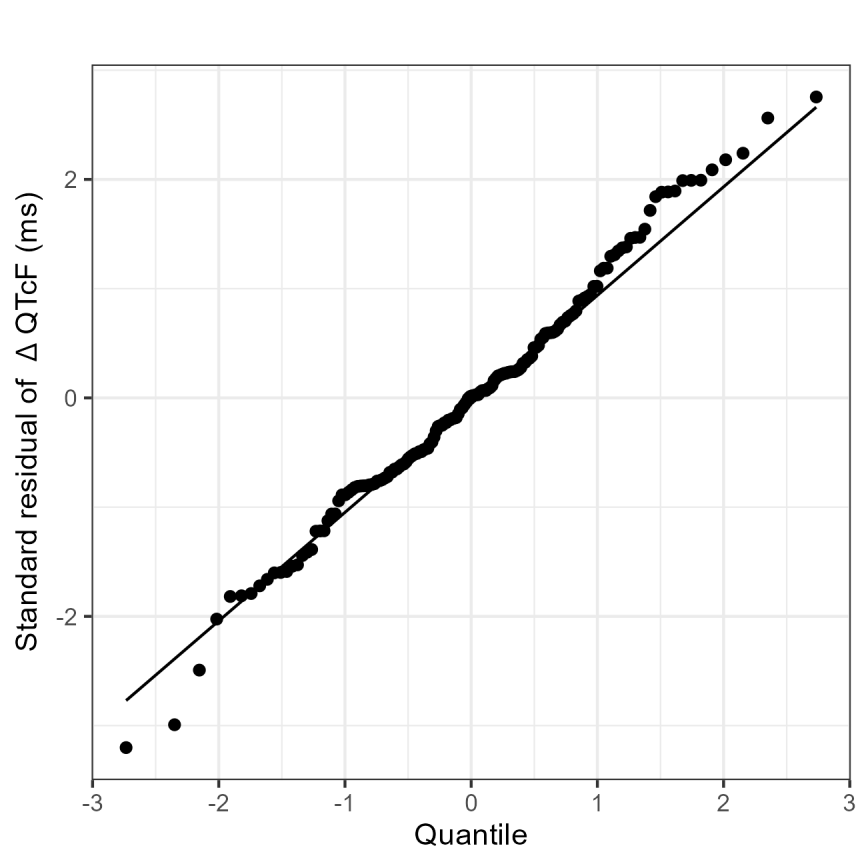 |  |
